# Assessing the Impact of Vehicle Speed Limits and Fleet Composition on Air Quality Near a School

**DOI:** 10.3390/ijerph16010149

**Published:** 2019-01-08

**Authors:** Jiayi Tang, Aonghus McNabola, Bruce Misstear, Francesco Pilla, Md Saniul Alam

**Affiliations:** 1 Department of Civil, Structural and Environmental Engineering, Trinity College Dublin, College Green, Dublin D02 PN40, Ireland; amcnabol@tcd.ie (A.M.); bmisster@tcd.ie (B.M.); Saniul.Alam@jacobs.com (M.S.A.); 2 Global Centre for Clean Air Research, Department of Civil and Environmental Engineering, Faculty of Engineering and Physical Sciences (FEPS), University of Surrey, Guildford GU2 7XH, UK; 3 Department of Planning and Environmental Policy, University College Dublin, Belfield, Dublin D04 V1W8, Ireland; francesco.pilla@ucd.ie; 4 Jacobs Engineering Group Inc., Dublin D04 R2C5, Ireland

**Keywords:** traffic emissions, speed limit, vehicle fleet, air pollution, school children

## Abstract

Traffic is a major source of urban air pollution that affects health, especially among children. As lower speed limits are commonly applied near schools in many cities, and different governments have different policies on vehicle fleet composition, this research estimated how different speed limits and fleet emissions affect air quality near a primary school. Based on data of traffic, weather, and background air quality records in Dublin from 2013, traffic, emission, and dispersion models were developed to assess the impact of different speed limits and fleet composition changes against current conditions. Outside the school, hypothetical speed limit changes from 30 km/h to 50 km/h could reduce the concentration of NO_2_ and PM_10_ by 3% and 2%; shifts in the fleet from diesel to petrol vehicles could reduce these pollutants by 4% and 3% but would increase the traffic-induced concentrations of CO and Benzene by 63% and 35%. These changes had significantly larger impacts on air quality on streets with higher pollutant concentrations. Findings suggest that both road safety and air quality should be considered when determining speed limits. Furthermore, fleet composition has different impacts on different pollutants and there are no clear benefits associated with incentivising either diesel or petrol engine vehicles.

## 1. Introduction

Research has shown significant associations between traffic pollution and adverse human health effects related to lung, heart, psychological, and other body systems (e.g., increasing lung cancer, heart disease, dementia, and other health problems) [1,2,3,4,5,6,7,8,9,10,11]. Air pollution is a major environmental problem that causes 6.4 million premature deaths worldwide per year, which is 72% out of 9 million deaths from all types of pollution [12]. Children in particular have been noted to be at high risk of pollution-related disease. Even very low-level exposures to air pollutants during windows of developmental vulnerability can result in disease, disability, and death in childhood and in later life [13]. Traffic related air pollution is not only linked to worsening existing health problems in children, but is also connected with the development of diseases, such as asthma [14]. Traffic related air pollution is harmful to the development of children’s nervous system, causing neurodegeneration, neuro-inflammation, and problems connected to cognition [15,16,17]. Many other problems such as allergy and autism spectrum disorders have been found to be associated with traffic air pollution [18,19]. 

In Europe, the transport sector contributes approximately 15% of Particulate Matter (PM) emissions and 41% nitrogen oxides (NO_X_) emissions [20], and thus poses a significant threat to children. In Ireland, road transport contributed 46% of NO_X_, 26% of PM_2.5_, and 9% of PM_10_ in 2014 [21,22].

Many strategies have been investigated which aim at addressing the aforementioned health risks from transport sources and improving air quality. These have involved traffic management strategies, vehicle fleet composition upgrades, land use, and infrastructure optimisation. Traffic management strategies include implementing road or congestion pricing, setting up low emission zones, executing vehicle operating restrictions, optimising traffic signal timing, changing speed limits, encouraging eco-driving, providing pedestrian and bike facilities, and more. A recent review found some limited evidence suggesting that these strategies can reduce total traffic emissions or improve local air quality [23]. For example, increasing the number of electric vehicles in the fleet has been shown to reduce the emissions of both CO_2_ and PM_2.5_ [24]. However, Tang et al. [25] illustrated that traffic management strategies implemented in Dublin, Ireland, including changes to the heavy goods vehicle management and road infrastructure, had both positive and negative impacts on air pollution and greenhouse gas (GHG) emissions. Alam & McNabola [26,27] highlighted the limitations and potential negative impacts of Eco-driving on fleet-wide emissions. Ghafghazi & Hatzopoulou [28] found that traffic calming schemes (speed bumps) could increase both NO_2_ and NO_X_ concentrations. In addition, low emissions zones have been found to bring about positive effects on reducing PM_10_ and NO_2_ concentrations in Germany [29], whilst it had no clear effect on PM_10_ and NO_2_ concentrations elsewhere [30]. As such, the impact of traffic management strategies on air quality is not always clear and requires careful investigation. Moreover, research has found that both positive and negative impacts can be brought about by similar strategies [23]. It was suggested that the impact of similar strategies may vary from case to case. Therefore, the impacts of traffic management should be scrutinized in each case. 

Since it is evident that traffic air pollution is harmful to human health, especially in children, the impact of traffic management strategies on air quality near schools is worthy of investigation. Reductions in speed limits near schools are commonly implemented for traffic safety reasons, yet the air quality impact of this measure is unclear and not often considered. 

Traffic conditions are important for the accuracy of evaluating traffic management strategies. In this paper, we utilized a traffic model and an emission and dispersion model to estimate the air quality conditions in the vicinity of a school in Dublin (Ireland) across a particular year (2013, see below for justification), based on traffic count records, background pollutant concentrations, meteorological data, and the topography of the area near the school. We evaluated a number of traffic management strategies that could have impacts on air quality near the school (i.e., different speed limit settings and fleet changes). Predicted air quality impacts of traffic management changes were compared against current conditions. 

Assessing the effect of these elements helps increase the awareness of policy makers as to the extent to which these strategies influence air quality, and enables informed evaluation of whether it is worthwhile to apply these strategies to improve children’s health. 

## 2. Research Methodology

**Figure 1:**
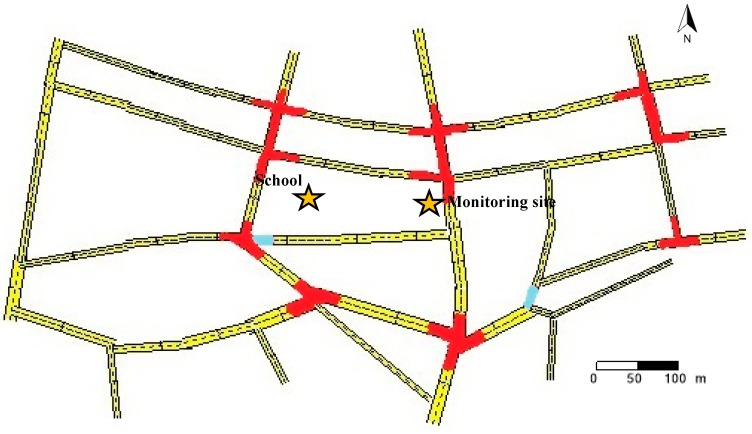
Modelled area. (Calibrated roads (red) and validated roads (cyan)).

A modelling chain approach was applied to evaluate the traffic and air quality conditions near a school, and to estimate potential impacts of changes in speed limits and fleet composition on air quality. The year 2013 was chosen for the analysis as it had the most complete and up to date dataset available. The area selected for the case study contained a primary school (children aged 4–11) located in Dublin City centre in Ireland (Figure 1). Road-side air quality monitoring has been conducted 200 m to the east of the school as part of the fixed site air quality monitoring network within Dublin [31]. Section 2.1 discusses the model development and the data used for the estimation of air quality. Section 2.2 describes the hypothetical scenarios that were used to estimate the impact of speed limit and fleet composition changes. 

### 2.1. Model Development

**Figure 2:**
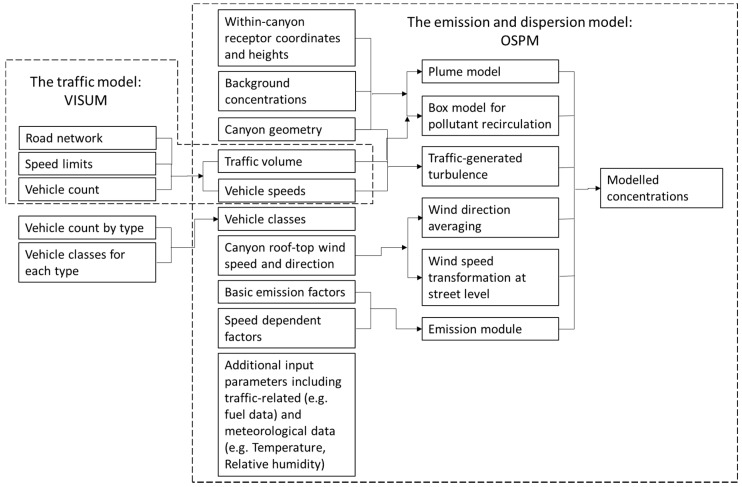
Schematic representation of the principal modules of VISUM & OSPM modelling chain. (Adapted from: Aquilina & Micallef, 2004 [35]).

The modelling chain comprised a traffic model and an emission and dispersion model (See Figure 2). The size of the modelled area was approximately 40 hectares. A primary school was located at the centre of the modelling area. This area was chosen to represent the vicinity of the school, and the traffic and air quality conditions around the school. Distance–decay studies on traffic air pollutant concentrations have found that motorways have impacts on concentrations of NO_X_ and PM typically up to 200m away from the roadside [6]. Roads in the city centre generally have lower traffic volumes than motorways, and a street canyon topography is often present, which concentrates the air pollution and prevents it from dispersing freely. Therefore, roads in the city centre have a smaller area of influence compared to motorways. In addition, many pupils of the school live within the modelled area, and thus its air quality is crucial for their health. 

The traffic model was developed in VISUM [32] and was used to assess the traffic volume and vehicle speed on the road network. The traffic condition outputs from the traffic model were used as an important input to the emission and dispersion model, which was developed using the Operational Street Pollution Model (OSPM) [33]. The methodology for emission calculations and emission factors for different types of vehicles that were applied in OSPM were based on the European Emission Model COPERT4 [34]. The study focused on hourly pollutant concentration predictions across a full year, and the implementation of a traffic assignment model (VISUM) and emission calculation method based on hourly average speed (COPERT4) complied with the objective of the study. The details of the development of the traffic model, and the emission and dispersion model, are explained in the following subsections. 

#### 2.1.1. Traffic Model

The flow chart of the traffic model is illustrated as part of the modelling chain in Figure 2. The traffic model estimated traffic volume and traffic speed based on speed limit and traffic condition inputs on each road in the network. VISUM was chosen as the traffic model because of the flexibility to adjust traffic volume based on traffic count, and the ability to use Python script to control the modelling process. 

The road network was developed in VISUM to represent the roads of the modelling area shown in Figure 1. The original travel demand was presented as an OD matrix for the modelled area and was derived from a traffic model for the city, developed by Tang et al. [25]. This traffic model for the city was obtained by extracting Dublin city from the National Transport Model (NTpM) of Ireland and was calibrated with the annual average traffic count records of 2013 for roads in Dublin. 

The original OD matrix was then calibrated with traffic count data throughout the year 2013 to obtain a more precise travel demand. The traffic count data were derived from hourly traffic counter data from Dublin City Council (DCC). The traffic conditions for weekdays during ten months of 2013—excluding July and August—were modelled to represent the days when the traffic pollution in the modelled area were of most relevance to pupils in the school (pupils did not attend school during weekends or in the summer holiday months of July and August). The traffic model was calibrated using one week of hourly traffic data from every two-month period in 2013. Analysis was conducted using a Python script because of the large amount of data involved in hourly travel data across a full year. The traffic data involved in the calibration were chosen to be representative of various traffic conditions throughout the school year (i.e., they included all weekdays, rainy and sunny days, spring, autumn, and winter periods). 

During the calibration process, the original OD matrix was assigned to roads to obtain traffic volumes using a user-equilibrium assignment approach. Then the assigned volumes were compared to traffic counts and adjustments were made to OD matrices to obtain more accurate results. This step of the assignment of the OD matrix to roads, the comparison between the assigned volumes and traffic counts, and the adjustment of the OD matrix was repeated several times to obtain an accurate matrix to reflect the traffic condition for each hour corresponding to traffic count data. In this process, traffic count data had to be set as an input to VISUM for each hour at several roads. A second Python script was established to conduct this iterative process automatically. A validation process of the traffic volume was performed after the calibration process. Hourly traffic count data for 600 h of 37 roads were derived from DCC, among which 35 were chosen for calibration and the remaining 2 were reserved for validation. Roads that were calibrated and validated are indicated in Figure 1 with red and cyan colours, respectively. 

The Geoff Havers (GEH) statistic was chosen to evaluate the validation. A guidance regulated in the Design Manual for Roads and Bridges (DMRB) in the United Kingdom was chosen as the criteria for the validation [36]. The calculation of GEH and the criteria is summarized in Table 1. 

During the assignment process, the traffic volume on each road was determined not only by the OD matrices but also influenced by the relationship between traffic volume and speed, which was represented by a volume-delay function (VDF). A logistic form of the VDF was adopted as it was found to be more accurate when applied to the prediction of speed measurements in this study, as shown in Equation (1):

(1) $$t_{cur}=t_{0}+\frac{a}{1+f\cdot e^{b-d\cdot sat}}$$

where $sat=\;\frac{q}{c\cdot q_{max}}$, *t_cur_* represents travel time modelled, *t*_0_ represents the free flow travel time, *q* represents the traffic volume, and *q_max_* represents the road capacity. The values of the parameters in Equation (1) are listed below:

a=0.1, b=9, c=2, d=28, f=1

The comparison between the modelled and recorded traffic average speeds and the result of traffic volume validation are presented in Section 3.1.

#### 2.1.2. Dispersion and Emission Model

The flow chart of the emission and dispersion model is also shown in Figure 2. The traffic emissions were calculated in OSPM based on the predicted traffic flow in streets (vehicles/hour), the traffic speed, and the emission factors at certain speeds for particular types of vehicles (g/vehicle/km). Only the exhaust emissions were considered in this study.

The fleet composition was derived from traffic count data and the Irish national fleet composition. Fleet proportions for each category (e.g., cars, buses, and trucks) were derived from the count data (across 2013) in Dublin city centre. Within each category, the percentages for each vehicle sub-category (e.g., petrol > 2.0 l with PC Euro 3 and Diesel > 2.0 l with PC Euro 4) were assumed to be in proportion with the Irish national fleet composition [37], following the same methodology applied by [25]. Summarized fleet data are presented in Table 2. Further details of the fleet data are given in Table A1 in the Appendix A. A 1% composition of benzene in petrol was assumed, in line with the EU regulation [38]. 

Information on traffic conditions, which included the traffic volume and speed for each hour of the year and fleet composition information, was developed using a third Python script to gather information from the traffic model, and then formatted and inputted into OSPM.

Besides emissions, other inputs to OSPM that influenced pollutant concentrations included road and building geometry information, weather information and background pollutant concentration. These data were obtained from Google Maps, the Irish meteorological service, and the air quality monitoring network of the Environmental Protection Agency of Ireland, respectively. A summary of these elements is shown in Table 3. 

In order to facilitate a holistic assessment of traffic emissions, the major harmful pollutants that originate from traffic were included, namely NO_X_, PM_10_, PM_2.5_, CO, and Benzene. Regarding NO_X_, a special focus was given to the modelling concentrations of NO_2_ (in the Modelling results section), as NO_2_ is a major pollutant concerning public health. The modelled concentrations consisted of a background concentration component and a component arising from the local traffic in each road. Whilst the impacts of speed limit and fleet composition changes on the concentrations of NO_X_, PM_10_, PM_2.5_, CO, and Benzene on each road were estimated, background concentrations were only available for PM_10_ and NO_X_. Therefore, while total concentrations of NO_X_ and PM_10_ were modelled, the traffic-induced concentrations of PM_2.5_, CO, and Benzene were modelled.

In OSPM, concentrations of traffic emissions were calculated using a combination of a plume model for the direct contribution of traffic pollution, and a box model for the recirculating part of the pollutants in the street, taking street canyon geometry into account. The receptors (the points of which pollutant concentrations were estimated) were set to be on the building face in the centre of each street section at a height of 1.2 m. These street sections, with lengths of 80 m to 120 m, constituted the streets shown in Figure 1. The reason that the receptor height was set to 1.2 m was because the age of children in the school in the modelling area was in the range of 4 to 11, and the average height for children in this age range is around 1.2 m in Ireland [39]. The receptor height of the street on which the air quality monitoring site was located was set to 2m, the same height as the as the monitor. 

### 2.2. Scenarios 

The air quality conditions estimated from the traffic counts in 2013 near the school were considered as the baseline scenario. The air quality of hypothetical speed limit changes and fleet composition changes were evaluated as alternative scenarios and compared to the baseline. The baseline scenario and six hypothetical scenarios are summarized in Table 4. 

At present, diesel cars account for only 3% of total passenger vehicles in the United States and less than 1% in China, whereas this is about 50% in Europe, and it is 45% in Ireland [37,40]. Compared to petrol cars, diesel cars have better fuel economy than petrol-powered cars, thus their emission of CO_2_ may be lower. However, diesel remains a major source of harmful pollutants (e.g., ozone-forming gases, including NO_X_ and PM) [40]. Therefore, in line with several recent proposals to reduce the prevalence of this source of air pollution in European cities [41], this paper compares the impact on air quality outside a school in an urban setting of converting diesel cars to comparable petrol cars. 

Also, Dublin City Council reduced the speed limit within the modelled area from 50 km/h to 30 km/h in 2009 because of road safety considerations within the city centre. This paper, therefore, also assesses the impact of the speed change by modelling the impact of two hypothetical speed limits on air quality.

## 3. Modelling Results

### 3.1. The Traffic Model

#### 3.1.1. Volumes and Speeds 

**Figure 3:**
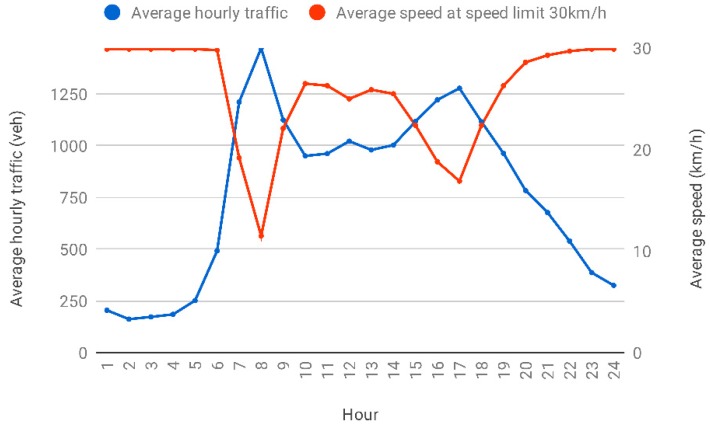
The average hourly traffic and average speed at the speed limit of 30 km/h at different hours of a day for all road segments across 2013.

The average modelled traffic volumes and speeds within the modelled area are summarised in Figure 3, showing the average hourly traffic data and average speed for all road segments within the baseline scenario (speed limits of 30 km/h) during different hours of the day. The average modelled speed at AM peak hour (8 to 9 a.m.) and PM peak hour (5 to 6 p.m.) of 12 km/h and 21 km/h was very similar to the measured average traffic speed in the city centre of 13 km/h and 19 km/h [42]. During off-peak hours, the average modelled speed returned towards the relevant speed limit. 

#### 3.1.2. Result of Validation for Traffic Volumes

A summary of validation results, using the GEH statistics approach, is presented in Table 5. The validation satisfied the DMRB criteria shown in Table 1, resulting in a robust model which represented the travel demand after being calibrated using the recorded volumes.

### 3.2. Dispersion Model Validation 

**Figure 4:**
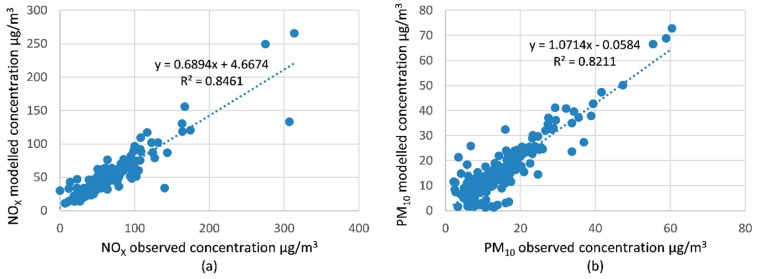
Modelled vs. observed concentrations for (**a**) NO_X_ and (**b**) PM_10_ daily average concentration in 2013.

Studies elsewhere have compared and validated local-scale emission and dispersion models (including OSPM) with in-situ measurements and have found a good fit with this modelling approach, especially for annual average concentrations [35]. Figure 4 shows the results of modelled NO_X_ and PM_10_ daily average concentrations compared against the observed concentrations in Dublin city centre. The modelled concentrations were acquired from the particular road on the network where the monitoring site was located, and also on the side of that road where the monitor was located. 

The modelled concentrations fitted the observed concentrations, with an R^2^ = 0.85 and 0.82 for NO_X_ and PM_10_, respectively. The modelled PM_10_ concentrations had good accuracy, with a slope of 1.07. However, the modelled NO_X_ data underestimated the measured concentration, where the slope was 0.69. Previous investigations have found similar underestimations of NO_X_ using the OPSM model [43]. 

### 3.3. The Effect of Speed Limit Changes

**Figure 5:**
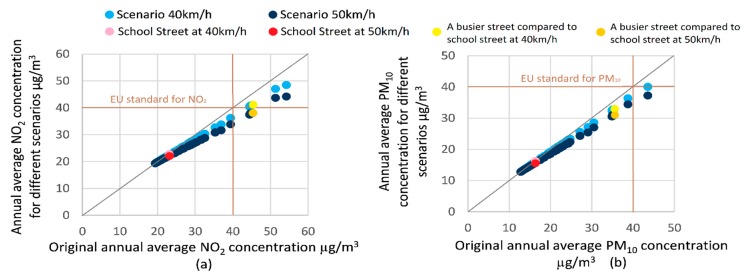
Predicted change in (**a**) NO_2_ and (**b**) PM_10_ total street concentrations on each road segment in the model domain for varying speed limits.

**Figure A1:**
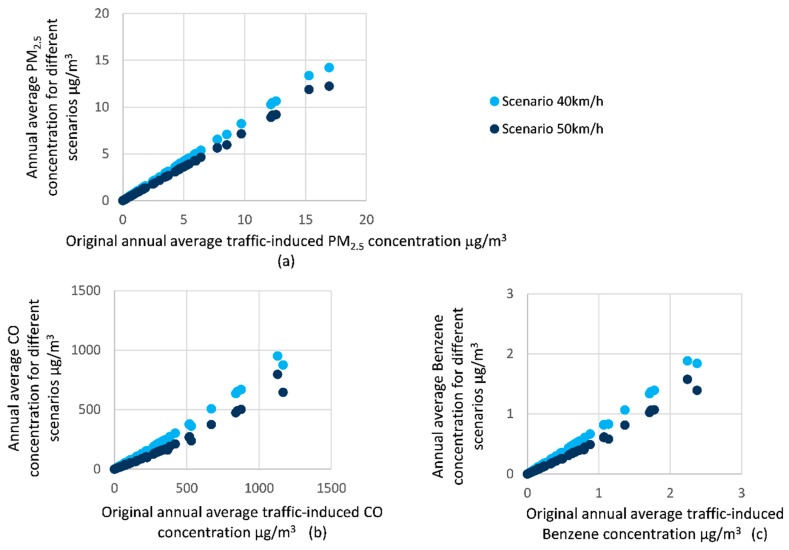
Predicted change in (**a**) PM_2.5_, (**b**) CO, and (**c**) Benzene traffic induced concentrations on each road segment in the model domain for varying speed limits.

The modelled impacts of speed limits on the total concentrations of NO_2_ and PM_10_ in each street section in the modelled area are summarised in Figure 5a,b. Each individual data point in Figure 5 represents the concentration on a specific road link for the speed limit change scenarios compared to the baseline scenario. The x-axis shows the baseline scenario concentration, and the y-axis shows the concentrations for two speed limit scenarios. A 1:1 relationship between the baseline scenario concentration and the speed limit change scenario is indicated with a grey diagonal line. A light red dot and a dark red dot highlight two speed limit change scenarios for the road on which the school is located. Traffic volumes, and consequently air pollution concentrations, on this street were less than for the majority of road links in the rest of modelled area. The EU air quality standards for annual NO_2_ and PM_10_ concentrations are also indicated with brown lines. The impact of speed limits on the traffic-induced concentrations of PM_2.5_, CO, and Benzene are summarised in Figure A1a–c, respectively, in the Appendix A. 

Figure 5 shows a decline in both NO_2_ and PM_10_ concentration for scenarios with hypothetical increases of speed limits from 30 km/h to 40 km/h and 50 km/h. The alteration of the speed limit from 30 km/h to 50 km/h could reduce NO_2_ by up to 18%, depending on the original concentration in the street in the baseline scenario. This action could also reduce PM_10_ by up to 15%, also depending on the original concentration. The general trend shows that NO_2_ and PM_10_ concentrations could be reduced further for the streets that originally had higher NO_2_ and PM_10_ concentration. Regarding the traffic-induced concentration, alteration of the speed limit from 30 km/h to 50 km/h could reduce these concentrations of NO_2_ and PM_10_ by 21% to 30% and 14% to 22%, respectively, depending on the street in question. As background concentration for different streets was assumed to be the same, the higher the NO_2_ and PM_10_ concentration was on the street, the higher the pollutant concentration that was generated by traffic, and therefore, the speed limit change strategy that influences traffic conditions would have a larger effect on the pollutant concentration. 

From Figure 5, for both NO_2_ and PM_10_, we can see that there were several streets where the original concentration was beyond the EU limit value, and this was predicted to reduce to below the limit as a result of the proposed speed changes. Therefore, the hypothetical speed limit changes modelled here would allow the pollutant concentrations on more streets to adhere to the EU air quality standards. 

As the school was located in a place with low traffic volumes and was thus less polluted, the effect of the speed change on air quality was relatively small compared to other streets with heavier pollution. Comparing the speed limit change impact in front of the school with a busier street (yellow dots shown in Figure 5), the predicted concentrations of NO_2_ and PM_10_ could only be reduced by a maximum of 3% and 2%, respectively, on the street in front of the school. This corresponded to a 22% and 15% reduction of traffic-induced NO_2_ and PM_10_. These figures could be reduced by 15% and 12%, respectively, on the street with higher original pollutant concentration (corresponding to 27% and 19% reduction of traffic-induced NO_2_ and PM_10_).

### 3.4. The Effect of Fleet Composition

**Figure 6:**
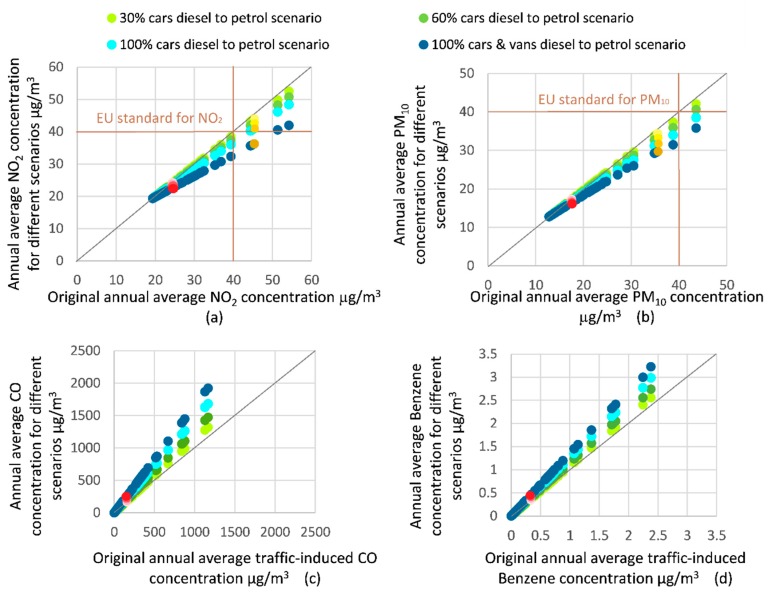
Predicted percentage change in (**a**) NO_2_ and (**b**) PM_10_ total street concentrations, and (**c**) CO and (**d**) Benzene traffic induced concentrations on each road segment in the model domain for varying fleet compositions.

**Figure A2:**
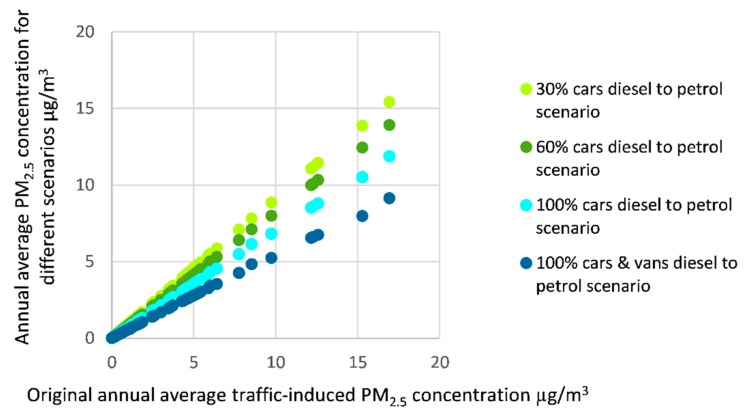
Predicted change in PM_2.5_ traffic induced concentrations on each road segment in the model domain for varying fleet compositions.

The impact of fleet composition on the total concentrations of NO_2_ and PM_10_ in each street is shown in Figure 6a,b, whilst the impact of fleet composition on the traffic-induced concentrations of CO and Benzene is shown in Figure 6c,d. Traffic-induced concentrations were estimated instead of total concentration of CO and Benzene because of the absence of background concentration information for these gases. The impact of fleet composition on the traffic induced concentrations of PM_2.5_ is shown in Figure A2 in the Appendix A. 

In Figure 6, the x-axis shows the baseline scenario concentration, and the y-axis shows the concentrations for four fleet composition change scenarios. Again, the grey diagonal line indicates the 1:1 relationship between the baseline scenario concentration and the fleet composition change scenario. Brown lines show the EU standards for annual NO_2_ and PM_10_ concentration. 

Figure 6a,b show declines in NO_2_ and PM_10_ concentration for the modelled fleet composition change scenarios. This demonstrates that increasing the proportion of petrol vehicles can reduce the NO_2_ and PM_10_ by up to 23% and up to 19%, depending on the street in question. This hypothetical strategy can reduce the traffic-induced NO_2_ and PM_10_ by up to 35% and up to 28%, respectively. For similar reasons as outlined above, the general trend for NO_2_ and PM_10_ concentrations was that they could be reduced by a greater amount for the streets that originally had higher concentrations. 

Among all these scenarios, including speed limit change scenarios, replacing all diesel cars and vans with petrol cars and vans had the greatest predicted impact on NO_2_ and PM_10_ concentration. The high original NO_2_ and PM_10_ concentration of several streets, which exceeded the EU limit value, as shown in Figure 6a,b, could be reduced to below the limit value with the shifting of diesel vehicles to petrol vehicles.

The effect of fleet composition change on the street in front of the school was relatively small compared to other streets with heavier pollution (Table 6). Regarding the reduction of the concentration of NO_2_ and PM_10_ on the street in front of school, the scenario of replacing all diesel cars and vans with petrol vehicles was the most efficient, reducing the NO_2_ and PM_10_ pollution concentrations by 4% and 3%, respectively. When compared to the 3% and 2% reductions found for the scenario of increasing the speed limit to 50 km/h, both interventions produced similar levels of impact. 

Regarding the traffic-induced concentrations of CO and Benzene, Figure 6c,d depict that, in general, increasing the proportion of petrol cars and vans would increase these concentrations appreciably. Thus, in contrast to NO_2_ and PM_10_, increasing the proportion of petrol vehicles would exacerbate the CO and Benzene pollution. By changing all the diesel cars and vans to petrol cars and vans, the traffic-induced concentration of CO would be increased by 65% at maximum. This strategy would also increase traffic-induced Benzene concentration by 36% at maximum. The scenario of changing diesel vehicles to petrol vehicles could increase the traffic-induced CO and Benzene concentration at the school by 63% and 35%, respectively.

## 4. Discussion 

This investigation showed that speed limits and fleet composition changes could have notable impacts on the air quality. These scenarios could potentially affect the air quality at the location of the school in question here, albeit to a lesser extent than they would affect the air quality along the more polluted streets away from the school.

Regarding speed limits, the hypothetical increase of speed limits from 30 km/h to 50 km/h was predicted to reduce the concentrations of all the pollutants examined here (i.e. NO_2_, PM, CO, and Benzene). Ghafghazi & Hatzopoulou [28] found that speed bumps that are used to reduce speeds could lead to an increase in both NO_2_ and NO_X_ concentrations in urban areas. Int Panis et al. [44] estimated that a speed limit change from 50 km/h to 30 km/h would increase NO_X_ and PM emissions by 2% to 5% and 3% to 8%, respectively, using the COPERT methodology. Carsten et al. [45] found that PM and NO_X_ would increase by 1% and 0.7% when the number of vehicles conforming to a speed limit of 30 mph was increased. These results are in line with the results of the present study. Some studies have also found a decrease in traffic emissions with speed limit changes from 50 km/h to 30 km/h in Belgium, using microsimulation [44,46]. To date there have been few ex-post studies that can support the conclusions made by the different analyses of speed limit changes, and clear-cut conclusions about the impact of these strategies on air quality do not exist [23].

The results of this study also showed that for the streets that experienced more severe air pollution, the changing of speed limits would have more impact on traffic-related air pollution. For example, EU limit values could be achieved in some streets where the original pollutant concentration exceeded the limit. Regarding the speed limit decrease from 50 km/h to 30 km/h introduced in 2009, this would not have affected the children significantly in terms of the air quality health impacts as the school within this study area is located along a less-busy, less-polluted street. However, this study does also indicate that, for a busy and heavily polluted area, a strategy of decreasing the speed limit could potentially lead to a reduction in air quality, with consequent implications for health.

In assessing the potential risks of vehicle speed to human health, it is necessary that all risks are taken into account. In this respect, it is perhaps worth pointing out that air pollution is responsible for a considerable amount of premature deaths worldwide. In Ireland in 2014, 193 people were killed in road traffic accidents [47], while in the same year, air pollution was associated with 1,510 premature deaths [48]. However, transport is only one of many contributors to this figure. Therefore, it does not necessarily suggest that the influence of air pollution on human health outweighs that of traffic accidents. On the other hand, in terms of the relationship of speed limit and car accidents, a review suggests that “studies of the effectiveness of school zone limits have generally found poor driver compliance, particularly when the limits are set very low, and no relationship between pedestrian crashes and the special limits” [49]. 

In this study, measured traffic count data and speed records were used in the calibration and validation of the model. This enabled the traffic strategies being assessed to be based on the traffic conditions of Dublin in 2013. The emission calculation method used in the study is based on average speeds, which, although complying with the objective and time-scale scope of the study, restricted the evaluation of the impacts of acceleration and deceleration on traffic emission. The driving cycles that are adopted to develop the COPERT emission factors include the condition of driving in real urban roads, which infers that these EFs incorporate the emission related to acceleration and deceleration to some extent. However, changes that may be brought about in the amount of acceleration and deceleration occurring as a result of changes in speed limits are not fully captured using this approach. In order to quantify the instantaneous impacts of speed limit changes on emissions in more detail in future, a traffic micro-simulation would be required. However, such micro-simulations are often limited in their size of study area and length of time scale which can be covered, limiting the results of such investigations in contrast to the current approach. The current approach was based on the average speed in every hour of 2013 for the entirety of Dublin city, and as such was focused on the longer-term impacts of these traffic strategies over a large area. 

The health impact of the traffic emissions resulting from reducing speed limits should be taken into consideration, especially for schools located on busy roads. In contrast, outside an urban setting, road transport may contribute significantly less than other sources of air pollution to total exposure, and in such circumstances a speed limit reduction would be especially beneficial.

As petrol and diesel vehicles are still dominant in the fleet, and will be for some time, and there is no alternative that can replace these two types of vehicles worldwide in the short-term, it is worthwhile to evaluate these two types of vehicles in terms of their impact on air quality. Also, the emission change estimation of the fleet conversion to hybrid or electric is worthy of research in the future in line with the development and roll-out of this technology.

For different air pollutants, the effects of the changes of speed limit and the fleet composition were different. Increasing the proportion of petrol vehicles could reduce the on-street concentration of NO_2_ and PM, but this strategy would lead to a rise in the concentration of CO and Benzene. Thus, although the most significant modelling result for reducing the pollution of NO_2_ and PM was obtained by replacing all diesel cars and vans with petrol cars and vans, when considering the pollution of CO, benzene, and other pollutants that were not covered by our paper, this strategy appears inconclusive, with positive and negative impacts. Policy makers should take as many pollutants as possible into account in order to make a full assessment of the advantages and disadvantages of using petrol and diesel cars. The results of this study highlight that the incentivisation of one fossil fuel type over another has advantages and disadvantages. The negative impacts of dieselisation of the European vehicle fleet are well publicized [50]. However, a similar incentivisation of petrol engines would have resulted in problems of a different nature. Determining which policy is effective in tackling traffic-related health impacts should involve a health impact assessment to quantify the impact of increases and decreases in different pollutants with different levels.

Similar to the effect of changing speed limits, for the streets that experienced more severe air pollution, the strategies of increasing the proportion of petrol cars were more effective in dealing with NO_2_ and PM pollution. The improvement in air quality of implementing different traffic management strategies depends on how much the traffic contributes to the air pollution. Therefore, cities with severe traffic pollution will receive larger benefits when applying these traffic air pollution reduction strategies than those found in Dublin, where air quality is relatively good. 

## 5. Conclusions

This study analysed several strategies and related factors that could affect air pollution in a city centre near a school. Real traffic count data were applied and several vehicle fleet and speed limit scenarios were modelled. The results showed that both strategies can influence the traffic-induced air pollutant concentrations significantly. Changing diesel cars and vans to petrol cars and vans had both advantages and disadvantages; this strategy would lead to benefits in terms of reducing NO_2_ and PM pollution, but would increase the pollution from CO and Benzene. Thus, it is overly simplistic to provide incentives for using either petrol or diesel vehicles. Policy makers should seek to strike an appropriate balance of both in the fleet at present. Looking to the future, strategies should be implemented that are aimed at phasing out both types of vehicles and replacing these with e.g. electric vehicles or hybrid vehicles.

Decreasing speed limits near a school, although justified in terms of road safety, was shown to have potentially negative impacts in terms of air quality, especially for streets that are heavily polluted; thus, these impacts should be considered and balanced with the benefits to safety brought about by reducing the speed limit. 

## Figures and Tables

**Table 1:** Validation criteria (Adapted from U.K. DMRB, [36]). In GEH calculation formula, M is the hourly traffic volume from the traffic model and C is the real-world hourly traffic count.

Measure	Criteria	Guideline
$GEH=\sqrt{\frac{2{\left(M-C\right)}^{2}}{M+C}}$	GEH < 5	>85% of cases

**Table 2:** Fleet data for each category and fuel type for different fleet change scenarios.

Category	% of Fleet	Fuel Type	Percentage in Each Category by Scenario (with Scenario Number Shown in Table 4)				
			Baseline	30% Diesel Cars to Petrol (iv)	60% Diesel Cars to Petrol (v)	100% Diesel Cars to Petrol (vi)	100% Diesel Cars and Vans to Petrol (vii)
Passenger car	82%	Petrol	63%	74%	85%	100%	100%
		Diesel	37%	26%	15%	0%	0%
Van	12%	Petrol	0.30%	0.30%	0.30%	0.30%	100.00%
		Diesel	99.70%	99.70%	99.70%	99.70%	0%
Truck	1%	Petrol	0	0	0	0	0
		Diesel	100%	100%	100%	100%	100%
Bus	5%	Petrol	0	0	0	0	0
		Diesel	100%	100%	100%	100%	100%

**Table 3:** Input data for background concentration, building geometry and weather condition.

Input Type	Element	Descriptive Statistics		Notes
		Mean	Standard Deviation	
Background concentration	PM_10_	13.58 µg/m^3^	9.43	Data source: PM_10_ monitoring site located at inner suburb in Dublin
	NO_X_	28.06 µg/m^3^	38.37	Data source: NO_X_ monitoring site located at a park in Dublin
Building geometry	Building height/road width (H/W)	0.98	0.41	Data source: Google Maps
Weather condition	Wind speed	5.63 m/s	2.86	Data source: the Irish meteorological service. Wind direction: Prevailing winds are westerly and south-westerly winds
	Temperature	9.52 °C	5.46	
	Relative humidity	0.82	0.12	

**Table 4:** Scenarios information.

Scenario Type	Scenario	Notes
Baseline	i. 30 km/h with Irish national fleet composition in 2013	Reflection of the actual condition of 2013
Speed limit	ii. 40 km/h iii. 50 km/h	Baseline fleet composition was applied; speed limit was changed for these scenarios.
Fleet composition	iv. 30% of diesel cars converting to petrol cars v. 60% of diesel cars converting to petrol cars vi. 100% of diesel cars converting to petrol cars vii. 100% of diesel cars and vans converting to petrol vehicles	30 km/h speed limit was applied; detailed percentages of petrol and diesel vehicles for these scenarios are shown in Table 2 with the scenario number corresponding to each scenario number in this table.

**Table 5:** Summary of the result of traffic volume validation.

Validation	# of Links and Turns	# of Hours	# of Cases	# of Cases with GEH <5	% of Cases with GEH <5
	2	600	1200	1041	87%

**Table 6:** The impact of fleet composition changes on the concentration of NO_2_ and PM_10_ comparison between the street in front of the school (indicated by red dots in Figure 6) and a street with high original pollutant concentration (indicated by yellow dots in Figure 6).

Scenarios		Street in Front of the School	A Street with High Original Concentration
Original concentration	NO_2_	22 µg/m^3^	45 µg/m^3^
	PM_10_	14 µg/m^3^	35 µg/m^3^
Concentration with 30% diesel changing to petrol cars (change in traffic-induced concentration)	NO_2_	−1% (−5%)	−3% (−5%)
	PM_10_	−1% (−4%)	−3% (−5%)
Concentration with 60% diesel changing to petrol cars (change in traffic-induced concentration)	NO_2_	−2% (−10%)	−6% (−10%)
	PM_10_	−1% (−8%)	−6% (−10%)
Concentration with 100% diesel changing to petrol cars (change in traffic-induced concentration)	NO_2_	−2% (−16%)	−9% (−17%)
	PM_10_	−2% (−14%)	−11% (−17%)
Concentration with 100% diesel changing to petrol cars and vans (change in traffic-induced concentration)	NO_2_	−4% (−35%)	−20% (−35%)
	PM_10_	−3% (−22%)	−16% (−26%)

## Appendix A

**Table A1:** Irish fleet data in 2013 (source: Duffy et al., 2015).

Sector	Subsector	Technology	Vehicle # in 2013
Passenger Cars	Gasoline 0.8–1.4 l	PRE ECE	0
Passenger Cars	Gasoline 0.8–1.4 l	ECE 15/00-01	0
Passenger Cars	Gasoline 0.8–1.4 l	ECE 15/02	0
Passenger Cars	Gasoline 0.8–1.4 l	ECE 15/03	0
Passenger Cars	Gasoline 0.8–1.4 l	ECE 15/04	949
Passenger Cars	Gasoline 0.8–1.4 l	Open Loop	0
Passenger Cars	Gasoline 0.8–1.4 l	PC Euro 1–91/441/EEC	7739
Passenger Cars	Gasoline 0.8–1.4 l	PC Euro 2–94/12/EEC	201,061
Passenger Cars	Gasoline 0.8–1.4 l	PC Euro 3–98/69/EC Stage2000	276,634
Passenger Cars	Gasoline 0.8–1.4 l	PC Euro 4–98/69/EC Stage2005	251,944
Passenger Cars	Gasoline 0.8–1.4 l	PC Euro 5–EC 715/2007	39,297
Passenger Cars	Gasoline 0.8–1.4 l	PC Euro 6–EC 715/2007	0
Passenger Cars	Gasoline 1.4–2.0 l	PRE ECE	0
Passenger Cars	Gasoline 1.4–2.0 l	ECE 15/00-01	0
Passenger Cars	Gasoline 1.4–2.0 l	ECE 15/02	0
Passenger Cars	Gasoline 1.4–2.0 l	ECE 15/03	0
Passenger Cars	Gasoline 1.4–2.0 l	ECE 15/04	504
Passenger Cars	Gasoline 1.4–2.0 l	Open Loop	0
Passenger Cars	Gasoline 1.4–2.0 l	PC Euro 1–91/441/EEC	4434
Passenger Cars	Gasoline 1.4–2.0 l	PC Euro 2–94/12/EEC	106,786
Passenger Cars	Gasoline 1.4–2.0 l	PC Euro 3–98/69/EC Stage2000	146,907
Passenger Cars	Gasoline 1.4–2.0 l	PC Euro 4–98/69/EC Stage2005	136,307
Passenger Cars	Gasoline 1.4–2.0 l	PC Euro 5–EC 715/2007	21,812
Passenger Cars	Gasoline 1.4–2.0 l	PC Euro 6–EC 715/2007	0
Passenger Cars	Gasoline > 2.0 l	PRE ECE	0
Passenger Cars	Gasoline > 2.0 l	ECE 15/00-01	0
Passenger Cars	Gasoline > 2.0 l	ECE 15/02	0
Passenger Cars	Gasoline > 2.0 l	ECE 15/03	0
Passenger Cars	Gasoline > 2.0 l	ECE 15/04	33
Passenger Cars	Gasoline > 2.0 l	PC Euro 1–91/441/EEC	425
Passenger Cars	Gasoline > 2.0 l	PC Euro 2–94/12/EEC	7055
Passenger Cars	Gasoline > 2.0 l	PC Euro 3–98/69/EC Stage2000	9699
Passenger Cars	Gasoline > 2.0 l	PC Euro 4–98/69/EC Stage2005	9733
Passenger Cars	Gasoline > 2.0 l	PC Euro 5–EC 715/2007	1723
Passenger Cars	Gasoline > 2.0 l	PC Euro 6–EC 715/2007	0
Passenger Cars	Diesel 1.4–2.0 l	Conventional	182
Passenger Cars	Diesel 1.4–2.0 l	PC Euro 1–91/441/EEC	1452
Passenger Cars	Diesel 1.4–2.0 l	PC Euro 2–94/12/EEC	38,544
Passenger Cars	Diesel 1.4–2.0 l	PC Euro 3–98/69/EC Stage2000	132,268
Passenger Cars	Diesel 1.4–2.0 l	PC Euro 4–98/69/EC Stage2005	288,016
Passenger Cars	Diesel 1.4–2.0 l	PC Euro 5–EC 715/2007	160,045
Passenger Cars	Diesel 1.4–2.0 l	PC Euro 6–EC 715/2007	0
Passenger Cars	Diesel > 2.0 l	Conventional	26
Passenger Cars	Diesel > 2.0 l	PC Euro 1–91/441/EEC	209
Passenger Cars	Diesel > 2.0 l	PC Euro 2–94/12/EEC	5545
Passenger Cars	Diesel > 2.0 l	PC Euro 3–98/69/EC Stage2000	19,029
Passenger Cars	Diesel > 2.0 l	PC Euro 4–98/69/EC Stage2005	41,435
Passenger Cars	Diesel > 2.0 l	PC Euro 5–EC 715/2007	23,025
Passenger Cars	Diesel > 2.0 l	PC Euro 6–EC 715/2007	0
Passenger Cars	LPG	Conventional	0
Passenger Cars	LPG	PC Euro 1–91/441/EEC	72
Passenger Cars	LPG	PC Euro 2–94/12/EEC	57
Passenger Cars	LPG	PC Euro 3–98/69/EC Stage2000	57
Passenger Cars	LPG	PC Euro 4–98/69/EC Stage2005	57
Passenger Cars	LPG	PC Euro 5–EC 715/2007	0
Passenger Cars	LPG	PC Euro 6–EC 715/2007	0
Passenger Cars	Hybrid Gasoline < 1.4 l	PC Euro 4–98/69/EC Stage2005	0
Passenger Cars	Hybrid Gasoline 1.4–2.0 l	PC Euro 4–98/69/EC Stage2005	0
Passenger Cars	Hybrid Gasoline > 2.0 l	PC Euro 4–98/69/EC Stage2005	0
Light Commercial Vehicles	Gasoline < 3.5 t	Conventional	4
Light Commercial Vehicles	Gasoline < 3.5 t	LD Euro 1–93/59/EEC	28
Light Commercial Vehicles	Gasoline < 3.5 t	LD Euro 2–96/69/EEC	101
Light Commercial Vehicles	Gasoline < 3.5 t	LD Euro 3–98/69/EC Stage2000	236
Light Commercial Vehicles	Gasoline < 3.5 t	LD Euro 4–98/69/EC Stage2005	292
Light Commercial Vehicles	Gasoline < 3.5 t	LD Euro 5–2008 Standards	78
Light Commercial Vehicles	Gasoline < 3.5 t	LD Euro 6	0
Light Commercial Vehicles	Diesel < 3.5 t	Conventional	1441
Light Commercial Vehicles	Diesel < 3.5 t	LD Euro 1–93/59/EEC	10,951
Light Commercial Vehicles	Diesel < 3.5 t	LD Euro 2–96/69/EEC	39,482
Light Commercial Vehicles	Diesel < 3.5 t	LD Euro 3–98/69/EC Stage2000	92,220
Light Commercial Vehicles	Diesel < 3.5 t	LD Euro 4–98/69/EC Stage2005	113,834
Light Commercial Vehicles	Diesel < 3.5 t	LD Euro 5–2008 Standards	30,260
Light Commercial Vehicles	Diesel < 3.5 t	LD Euro 6	0
Heavy Duty Trucks	Gasoline >3.5 t	Conventional	24
Heavy Duty Trucks	Rigid ≤ 7.5 t	Conventional	108
Heavy Duty Trucks	Rigid <= 7.5 t	HD Euro I–91/542/EEC Stage I	287
Heavy Duty Trucks	Rigid <= 7.5 t	HD Euro II–91/542/EEC Stage II	1228
Heavy Duty Trucks	Rigid <= 7.5 t	HD Euro III–2000 Standards	2868
Heavy Duty Trucks	Rigid <= 7.5 t	HD Euro IV–2005 Standards	3541
Heavy Duty Trucks	Rigid <= 7.5 t	HD Euro V–2008 Standards	932
Heavy Duty Trucks	Rigid <= 7.5 t	HD Euro VI	0
Heavy Duty Trucks	Rigid 7.5–12 t	Conventional	129
Heavy Duty Trucks	Rigid 7.5–12 t	HD Euro I–91/542/EEC Stage I	345
Heavy Duty Trucks	Rigid 7.5–12 t	HD Euro II–91/542/EEC Stage II	1477
Heavy Duty Trucks	Rigid 7.5–12 t	HD Euro III–2000 Standards	3450
Heavy Duty Trucks	Rigid 7.5–12 t	HD Euro IV–2005 Standards	4258
Heavy Duty Trucks	Rigid 7.5–12 t	HD Euro V–2008 Standards	1121
Heavy Duty Trucks	Rigid 7.5–12 t	HD Euro VI	0
Heavy Duty Trucks	Rigid 12–14 t	Conventional	66
Heavy Duty Trucks	Rigid 12–14 t	HD Euro I–91/542/EEC Stage I	176
Heavy Duty Trucks	Rigid 12–14 t	HD Euro II–91/542/EEC Stage II	754
Heavy Duty Trucks	Rigid 12–14 t	HD Euro III–2000 Standards	1761
Heavy Duty Trucks	Rigid 12–14 t	HD Euro IV–2005 Standards	2173
Heavy Duty Trucks	Rigid 12–14 t	HD Euro V–2008 Standards	572
Heavy Duty Trucks	Rigid 12–14 t	HD Euro VI	0
Heavy Duty Trucks	Rigid 14–20 t	Conventional	43
Heavy Duty Trucks	Rigid 14–20 t	HD Euro I–91/542/EEC Stage I	114
Heavy Duty Trucks	Rigid 14–20 t	HD Euro II–91/542/EEC Stage II	487
Heavy Duty Trucks	Rigid 14–20 t	HD Euro III–2000 Standards	1137
Heavy Duty Trucks	Rigid 14–20 t	HD Euro IV–2005 Standards	1403
Heavy Duty Trucks	Rigid 14–20 t	HD Euro V–2008 Standards	370
Heavy Duty Trucks	Rigid 14–20 t	HD Euro VI	0
Heavy Duty Trucks	Rigid 20–26 t	Conventional	0
Heavy Duty Trucks	Rigid 20–26 t	HD Euro I–91/542/EEC Stage I	0
Heavy Duty Trucks	Rigid 20–26 t	HD Euro II–91/542/EEC Stage II	1
Heavy Duty Trucks	Rigid 20–26 t	HD Euro III–2000 Standards	3
Heavy Duty Trucks	Rigid 20–26 t	HD Euro IV–2005 Standards	4
Heavy Duty Trucks	Rigid 20–26 t	HD Euro V–2008 Standards	1
Heavy Duty Trucks	Rigid 20–26 t	HD Euro VI	0
Heavy Duty Trucks	Rigid 26–28 t	Conventional	0
Heavy Duty Trucks	Rigid 26–28 t	HD Euro I–91/542/EEC Stage I	0
Heavy Duty Trucks	Rigid 26–28 t	HD Euro II–91/542/EEC Stage II	1
Heavy Duty Trucks	Rigid 26–28 t	HD Euro III–2000 Standards	3
Heavy Duty Trucks	Rigid 26–28 t	HD Euro IV–2005 Standards	4
Heavy Duty Trucks	Rigid 26–28 t	HD Euro V–2008 Standards	1
Heavy Duty Trucks	Rigid 26–28 t	HD Euro VI	0
Heavy Duty Trucks	Rigid 28–32 t	Conventional	0
Heavy Duty Trucks	Rigid 28–32 t	HD Euro I–91/542/EEC Stage I	0
Heavy Duty Trucks	Rigid 28–32 t	HD Euro II–91/542/EEC Stage II	1
Heavy Duty Trucks	Rigid 28–32 t	HD Euro III–2000 Standards	3
Heavy Duty Trucks	Rigid 28–32 t	HD Euro IV–2005 Standards	4
Heavy Duty Trucks	Rigid 28–32 t	HD Euro V–2008 Standards	1
Heavy Duty Trucks	Rigid 28–32 t	HD Euro VI	0
Heavy Duty Trucks	Rigid > 32 t	Conventional	0
Heavy Duty Trucks	Rigid > 32 t	HD Euro I–91/542/EEC Stage I	0
Heavy Duty Trucks	Rigid > 32 t	HD Euro II–91/542/EEC Stage II	1
Heavy Duty Trucks	Rigid > 32 t	HD Euro III–2000 Standards	3
Heavy Duty Trucks	Rigid > 32 t	HD Euro IV–2005 Standards	4
Heavy Duty Trucks	Rigid > 32 t	HD Euro V–2008 Standards	1
Heavy Duty Trucks	Rigid > 32 t	HD Euro VI	0
Heavy Duty Trucks	Articulated 40–50 t	Conventional	0
Heavy Duty Trucks	Articulated 40–50 t	HD Euro I–91/542/EEC Stage I	0
Heavy Duty Trucks	Articulated 40–50 t	HD Euro II–91/542/EEC Stage II	1
Heavy Duty Trucks	Articulated 40–50 t	HD Euro III–2000 Standards	3
Heavy Duty Trucks	Articulated 40–50 t	HD Euro IV–2005 Standards	4
Heavy Duty Trucks	Articulated 40–50 t	HD Euro V–2008 Standards	1
Heavy Duty Trucks	Articulated 40–50 t	HD Euro VI	0
Heavy Duty Trucks	Articulated 50–60 t	Conventional	0
Heavy Duty Trucks	Articulated 50–60 t	HD Euro I–91/542/EEC Stage I	0
Heavy Duty Trucks	Articulated 50–60 t	HD Euro II–91/542/EEC Stage II	1
Heavy Duty Trucks	Articulated 50–60 t	HD Euro III–2000 Standards	3
Heavy Duty Trucks	Articulated 50–60 t	HD Euro IV–2005 Standards	4
Heavy Duty Trucks	Articulated 50–60 t	HD Euro V–2008 Standards	1
Heavy Duty Trucks	Articulated 50–60 t	HD Euro VI	0
Buses	Urban Buses Standard 15–18 t	Conventional	273
Buses	Urban Buses Standard 15–18 t	HD Euro I–91/542/EEC Stage I	190
Buses	Urban Buses Standard 15–18 t	HD Euro II–91/542/EEC Stage II	752
Buses	Urban Buses Standard 15–18 t	HD Euro III–2000 Standards	849
Buses	Urban Buses Standard 15–18 t	HD Euro IV–2005 Standards	596
Buses	Urban Buses Standard 15–18 t	HD Euro V–2008 Standards	369
Buses	Urban Buses Standard 15–18 t	HD Euro VI	0
Buses	Coaches Standard <= 18 t	Conventional	639
Buses	Coaches Standard <= 18 t	HD Euro I–91/542/EEC Stage I	445
Buses	Coaches Standard <= 18 t	HD Euro II–91/542/EEC Stage II	1765
Buses	Coaches Standard <= 18 t	HD Euro III–2000 Standards	1990
Buses	Coaches Standard <= 18 t	HD Euro IV–2005 Standards	1397
Buses	Coaches Standard <= 18 t	HD Euro V–2008 Standards	866
Buses	Coaches Standard <= 18 t	HD Euro VI	0
Mopeds	2-stroke < 50 cm^3^	Conventional	188
Mopeds	2-stroke < 50 cm^3^	Mop–Euro I	296
Mopeds	2-stroke < 50 cm^3^	Mop–Euro II	161
Mopeds	2-stroke < 50 cm^3^	Mop–Euro III	251
Mopeds	4-stroke < 50 cm^3^	Conventional	188
Mopeds	4-stroke < 50 cm^3^	Mop–Euro I	295
Mopeds	4-stroke < 50 cm^3^	Mop–Euro II	161
Mopeds	4-stroke < 50 cm^3^	Mop–Euro III	251
Motorcycles	2-stroke > 50 cm^3^	Conventional	3329
Motorcycles	4-stroke < 250 cm^3^	Conventional	699
Motorcycles	4-stroke < 250 cm^3^	Mot–Euro I	1099
Motorcycles	4-stroke < 250 cm^3^	Mot–Euro II	599
Motorcycles	4-stroke < 250 cm^3^	Mot–Euro III	932
Motorcycles	4-stroke 250–750 cm^3^	Conventional	5325
Motorcycles	4-stroke 250–750 cm^3^	Mot–Euro I	8368
Motorcycles	4-stroke 250–750 cm^3^	Mot–Euro II	4564
Motorcycles	4-stroke 250–750 cm^3^	Mot–Euro III	7100
Motorcycles	4-stroke > 750 cm^3^	Conventional	592
Motorcycles	4-stroke > 750 cm^3^	Mot–Euro I	930
Motorcycles	4-stroke > 750 cm^3^	Mot–Euro II	507
Motorcycles	4-stroke > 750 cm^3^	Mot–Euro III	789
